# Variables Associated With Body Image Concerns in Acromegaly Patients: A Cross-Sectional Study

**DOI:** 10.3389/fpsyg.2022.733864

**Published:** 2022-06-10

**Authors:** Xiaomei Zhang, Yanqing Li, Yueping Zhong, Ziheng Wang

**Affiliations:** ^1^Department of Neurosurgery, Affiliated Hospital of Nantong University, Nantong, China; ^2^Medical College, Nantong University, Nantong, China; ^3^Department of Surgery, Affiliated Hospital of Nantong University, Nantong, China

**Keywords:** acromegaly, body image, stigma, QoL, pituitary patients

## Abstract

**Objective:**

Acromegaly is often characterized by altered physical (including facial) appearance. However, there is little medical or psychological research on body image concerns in patients with acromegaly. The aim of this study was to identify factors influencing the body image distress in patients with acromegaly and to explore the possible effects of stigma on body image concerns.

**Design:**

Cross-sectional study.

**Methods:**

A total of 68 individuals with acromegaly were enrolled in the study. A total of 70 persons with nonfunctional pituitary adenomas were randomly recruited as a healthy control group. Using structured questionnaires, we explored perceived body image using the Body Image Concern Inventory. We also used the Hamilton Anxiety Scale, the Stigma Scale for Chronic Illness, the Brief Illness Perception Questionnaire, and the 36-Item Short-Form Health Survey to evaluate health-associated variables and to analyze factors that affect body image concerns in patients with acromegaly.

**Results:**

Of the 68 participants, 31 were men and 37 women (mean age ± standard deviation: 46.36 ± 12.47 years). The mean body image concern score was 47.49 ± 13.81 for patients with acromegaly and 21.10 ± 7.44 for patients with nonfunctional pituitary adenoma. The difference between the two groups was statistically significant. A multiple stepwise regression analysis showed that the related factors for body image distress were gender (*P* = 0.001), age at diagnosis (*P* = 0.01), and internalized stigma (*P* < 0.001, Adj. *R*^2^ = 0.756).

**Conclusions:**

People with acromegaly have substantial body image concerns, and these concerns are increased by the stigma associated with this disease; such concerns lead to poor quality of life (QoL). Physicians need to find better ways to control patients' hormone levels, and nurses should provide more information on how to improve body image or find ways to reduce patients' body image distress.

## Introduction

Pituitary adenomas (PA) account for 10–15% of all the intracranial tumors and are classified clinically into functional adenomas (if they cause excessive hormone generation) and nonfunctional adenomas, which, owing to a mass effect on the pituitary gland or adjacent tissues, are more likely to produce clinical symptoms (Alsumali et al., 2017). Acromegaly is caused by a type of PA and is a rare disorder (Holdaway and Rajasoorya, 1999). The disease is associated with severe disfigurement, characterized by numerous bone and soft tissue abnormalities. These are caused by an excess of growth hormone and include prognathism, acral growth, soft tissue swelling, macroglossia, hypertrichosis, and skin thickening. The early clinical manifestations of acromegaly are physical changes, universal visceral hypertrophy, and anterior lobe dysfunction. Late clinical manifestations of acromegaly include physical decline and the occurrence of secondary anterior pituitary dysfunction. Generally, the onset of acromegaly is insidious and the progression of the disease is slow (up to or >30 years) (Fleseriu et al., 2021). Childhood-onset growth hormone adenomas cause gigantism. Adult patients with the disease mainly show mass effect and endocrine disorders, such as coarse facial skin, headache, fatigue, sweating, backache, enlarged hands and feet, and increased hat and shoe size, but may also show symptoms and signs of diabetes and hyperthyroidism (Giustina et al., 2020). Such effects are usually insidious and may take up to 12 years to diagnose (Renehan et al., 2003; Melmed et al., 2018). Acromegaly diagnosis can be delayed for a variety of reasons, including disease rarity, poor rates of detection by nonexperts, subtle disfiguring changes over time, varying degrees of disease severity, and the mental status of patients (Sibeoni et al., 2019). The combination of abnormal hormone levels and delayed diagnosis means that patients can experience an abnormal body image for a long time.

Body image is defined as a person's perception of his or her own body and is particularly influenced by the awareness of other people's reactions to one's appearance, body shape, and body function. Body image has a substantial effect on an individual's emotions, thoughts, behaviors, and interpersonal relationships and is associated with physical and mental health (Cash et al., 2004, 2005). Previous studies have shown that major body image concerns can lead to serious psychological problems. Self-esteem has a mediating effect on the association among physical dissatisfaction, anxiety, and depression; a negative perception of body image reduces self-esteem and increases psychological distress (Duchesne et al., 2017; Vuotto et al., 2018). Various diseases can cause either temporary or permanent changes in the body image (Burg, 2016; Gholizadeh et al., 2019; Grilo et al., 2019; Henn et al., 2019; Song et al., 2020; Yamani Ardakani et al., 2020). Some body image changes can be easily remedied with medication or cosmetic surgery, but some can be difficult to change with the currently available medical technology. Previous research has shown that patients with acromegaly experience body image issues (Pantanetti et al., 2002; Roerink et al., 2015; Imran et al., 2016); for example, patients with acromegaly experience more paresthesia and accentuation of the body. Body image concerns can be associated with both mental health status and disease characteristics. However, the specific influencing factors of body image disturbances in patients with acromegaly remain unclear.

Acromegaly is often associated with altered physical as well as facial appearance. However, scarcely any research have been reported in the medical and psychological literature about the body image concerns in patients with acromegaly. This study is focused on finding the influencing and related factors of patients' body image distress and explore the possible effects of stigma on body image concerns. In addition, we used the Body Image Concern Inventory (BICI) to analyze if patients with acromegaly receiving long-term treatment experience more social dysfunction and psychological distress associated with body self-consciousness than an age- and gender-matched control group and whether this affects the quality of life (QoL).

## Methods

### Participants

Participants in the patient group were chosen according to the following inclusion criteria: (1) age ≥ 18 years; (2) clinical diagnosis of acromegaly; (3) disease duration > 3 months; (4) stable disease state, clear consciousness, and the ability to fully respond to the questions; (5) provision of written informed consent and agreement to take part in the survey. Patients with (1) profound dysphasia or aphasia; (2) underlying mental and psychotic conditions; and (3) severe comorbidities that have a substantial effect on QoL (such as cancer or acute heart failure) were excluded.

Participants in the nonfunctioning PA (NFPA) control group were chosen according to the following inclusion criteria: (1) a PA was evident on magnetic resonance imaging and (2) pituitary-related hormones were within the reference range. The exclusion criteria were as follows: (1) patient declined to participate in the study; (2) patient had difficulty finishing the evaluation; and (3) potential presence of tumors in other organs may have affected the evaluation of health status.

A research assistant provided potential participants with a thorough explanation of the study objectives, approaches, voluntary involvement, and right to confidentiality.

### Assessment Tools

Patients with acromegaly were asked to complete a series of standardized self-report questions as part of this study (described below).

### Sociodemographic and Clinical Characteristics

The following demographic factors were assessed: age at diagnosis; gender; marital status (married, unmarried); fertility status; residence (city, village); educational level (low, intermediate, high); profession (unemployed, farmer, manual worker, other occupation); economic status (high income, moderate income, low income, near/below poverty level); payment of medical expenses (self-pay, rural medical insurance, urban medical insurance); and smoking and alcohol use.

The following clinical variables were assessed: length of hospitalization (days); course of disease (years); body mass index (normal, overweight, obesity); prolactin (PRL) level; adrenocorticotropic hormone (ACTH) level; thyroid hormone level (low/normal/high); fasting blood glucose level; preoperative growth hormone level; postoperative growth hormone level; tumor size; the presence of other diseases; recurrence; comorbidity; and hormone therapy after discharge.

### Measures of Self-Report Variables

#### Body Image Concern Inventory

Littleton et al. (2005) developed the BICI. Possible scores on the English version of the BICI range from 19 to 95. The BICI is an effective and useful tool for determining the level of dysmorphic concern. Participants rate how frequently they experience the stated emotions or have undergone the specified actions for each of 19 items. Responses are on a 5-point rating scale ranging from 1 (never) to 5 (always). A total of 1,231 young Chinese people were asked to complete the Chinese BICI. Cronbach's alpha showed a total scale reliability of 0.92, and a test–retest reliability of 0.73 over 6 months (Wang et al., 2020).

#### Hamilton Anxiety Scale

The HAMA scale has been extensively used to evaluate anxiety symptoms. The HAMA comprises 14 questions with 5 possible responses to each question: 0 (never), 1 (mild), 2 (moderate), 3 (severe), or 4 (extremely serious). The total HAMA score is categorized as follows: no anxiety (score 0–6), mild anxiety (score 7–13), moderate anxiety (score ≥ 14), severe anxiety (score ≥ 21), and extreme anxiety (score ≥ 29). Previous research has demonstrated that the HAMA is a reliable and accurate measure of psychological state (Maier et al., 1988).

#### Stigma Scale for Chronic Illness

The SSCI was developed by Rao et al. (2009) to assess the stigma about neurological diseases. The SSCI was first used in 511 patients with neurological diseases. The first 13 items inquire about the respondent's emotions, such as self-blame, anxiety, and embarrassment, and are referred to as felt stigma. The following 11 questions are on enacted stigma and inquire about other people's behavior toward the respondent, such as staring, avoiding interaction, and being rude. Each item is evaluated on a scale of 1–5: 1 = never, 2 = rarely, 3 = sometimes, 4 = often, and 5 = always. The total score ranges from 24 to 120; higher scores indicate that the individual is more likely to be stigmatized.

#### The Brief Illness Perception Questionnaire

The BIPQ is a nine-item questionnaire that assesses nine aspects of how people feel about their diseases. Five items assess perceptual representations of disease: BIPQ-1, the prognostic effect of the disease; BIPQ-2, the course of the disease; BIPQ-3, the patient's control; BIPQ-4, evaluation of treatment control ability; and BIPQ-5, evaluation of disease symptom recognition ability. Two items assess the perception of disease: BIPQ-6 evaluates the level of disease care and BIPQ-8 assesses the severity of a disease's impact on the patient's mood. Item BIPQ-7 evaluates the patient's understanding of the disease. The final question, which is an open-ended question, asks patients to identify the top three factors that they think caused their illness. The first eight questions are graded on a scale of 0–10. Higher scores on these questions indicate positive illness perspectives; thus, the treatment control, personal control, and coherence questions are reverse-scored (Broadbent et al., 2006, 2015). Scores on the eight questions are summed to obtain an aggregate BIPQ score. A higher BIPQ score indicates a greater psychological burden of the disease (score range: 0–80). The reliability coefficient (Cronbach's α) of the Chinese version of the BIPQ was 0.77, and the reliability coefficient of each item was >0.6, which indicates that the scale has good internal reliability.

#### The 36-Item Short Form Health Survey

The 36-Item Short-Form Health Survey (SF-36) generates two summary scores and scores on the following eight subscales: role limitations due to physical health (4 items), physical functioning (10 items), fatigue/energy (4 items), role limitations due to emotional problems (3 items), social functioning (2 items), pain (2 items), general health (5 items), and emotional health (5 items). The SF-36 does not produce an overall score. High SF-36 scores indicate excellent overall health. We used the SF-36 Version 2 with a 4-week recall interval. Unlike other questionnaires that are condition specific, the SF-36 is a generic questionnaire. The domain scores range between 0 and 100; 0 indicates poor health and 100 indicates the greatest possible health. The SF-36 produces a mental component summary score and a physical component summary score. The SF-36 survey had high internal consistency reliability (Cronbach's alpha) of 0.93–0.78, which is higher than the suggested minimum of 0.70 (Li et al., 2003; van der Meulen et al., 2020).

### Data Collection Procedure

The following methods were used to collect the questionnaire data: self-completion, investigators' face-to-face inquiries, and telephone interviews. The investigator promptly checked each questionnaire after it was completed and scored the questions. During the data entry process, data were double checked to ensure the accuracy of data entry.

### Data Analysis

The scores for the acromegaly group and the NFPA group were determined using the independent sample *t*-test. The data were analyzed using IBM SPSS 26.0 statistical software. The values are expressed as number and (mean ± standard deviation). The following methods were used for the data analysis: (1) Values were analyzed using Pearson correlation analysis; (2) Values were analyzed using the independent sample *t*-test; and (3) Values were analyzed using a one-way analysis of variance. A multiple stepwise linear regression model was generated using SPSS to test whether the model was linear and whether the data were normally distributed, homogeneous, and independent, using the threshold (*P* < 0.3) as the screening criterion for entry into multiple stepwise linear regression analysis (Costa et al., 2015), and the results were considered significant when the *P*-value was <0.05 (two-sided). The results were analyzed using SPSS 26.0 and GraphPad Prism.

### Ethics Approval

The study conformed to the ethical standards of the Declaration of Helsinki. The hospital ethics committee authorized this research. Participants were informed that the information obtained in this study would remain strictly confidential. Before the investigation, all patients provided informed consent.

## Results

### Participants

Of the 89 patients we followed up, 68 patients with acromegaly were enrolled in the study. Of the patients admitted to our hospital over the last 10 years, 89 patients met the criteria for inclusion. Two of these patients died owing to severe complications. Eighteen patients declined to answer or could not be contacted, and one patient was unconscious after surgery and unable to answer questions. Therefore, 21 participants were excluded. All included participants completed the questionnaires (BICI, HAMA, SSCI, BIPQ, and SF-36) and attended an interview. Additionally, we randomly recruited 90 NFPA, who completed the BICI and the SF-36, and finally received 70 valid questionnaires.

### Characteristics of Included Patients

A total of 68 patients with acromegaly were enrolled (31 men and 37 women, aged 46.36 ± 12.47 years). The prior treatment growth hormone level in all patients was higher than normal, with an average level of 19.32 ± 11.49 ng/mL. The average level of PRL was 894.85 ± 2,672.75 mIU/L. The average level of ACTH hormone was 21.83 ± 22.17 pg/mL. Growth hormone levels returned to normal after treatment in 31 patients, while 37 patients still had growth hormone levels above normal, and the average level of postoperative growth hormone was 5.12 ± 7.07 ng/mL. In addition, a total of 70 patients with NFPA were included as a control group (35 men and 35 women, aged 49.77 ± 8.91 years). Age (*P* = 0.068) and gender (*P* = 0.668) did not significantly differ in the NFPA group and the acromegaly group. The demographic, clinical, and psychological characteristics of the included patients are further detailed in Tables 1, 2.

**Table 1:** Univariate analysis of demographic variables of body image concern in patients with acromegaly.

**Characteristics**	**Patients (*N*)**	**Total score of BICI**	**Statistical value**	***P-*value**
Age at diagnosis^†^	46.36 ± 12.47 (years)	–	−0.181	**0.139**
Gender^‡^			−0.917	**0.363**
Female	37	48.89 ± 13.73		
Male	31	45.81 ± 13.93		
Marriage^‡^			−3.086	**0.056**
Married	65	46.92 ± 13.81		
Unmarried	3	59.67 ± 6.51		
Whether fertility^‡^			0.019	0.986
Yes	62	47.50 ± 13.11		
No	6	47.33 ± 21.37		
Residence^‡^			−0.095	0.925
City	10	47.10 ± 10.45		
Village	58	47.55 ± 14.38		
Educational level^§^			0.904	0.410
Low	5	52.80 ± 3.96		
Intermediate	18	49.83 ± 11.32		
High	45	45.96 ± 15.21		
Smoke^‡^			0.324	0.747
Yes	10	48.80 ± 12.45		
No	58	47.26 ± 14.11		
Alcohol^‡^			−0.489	0.626
Yes	17	46.06 ± 13.48		
No	51	47.96 ± 14.01		
Payment of medical expenses^§^			3.897	**0.025**
Self-pay	2	26.50 ± 13.43		
Rural medical insurance	29	51.10 ± 10.72		
Urban medical insurance	37	45.78 ± 14.91		
Profession^§^			1.609	**0.196**
Unemployment	5	50.20 ± 12.96		
Farmer	16	50.50 ± 8.83		
Manual worker	42	44.90 ± 14.93		
Other occupations	5	56.80 ± 14.97		
Economic situation^§^			1.729	**0.170**
High income	2	35.50 ± 6.36		
Moderate income	45	46.00 ± 14.61		
Low income	18	53.00 ± 10.73		
Near/below poverty level	3	44.67 ± 14.57		

*BICI, Body Image Concern Inventory; NFPA, nonfunctioning pituitary adenomas*.

*Values are presented as number and mean ± standard deviation*.

† *Values are analyzed by Pearson correlation analysis*.

‡ *Values are analyzed by independent sample test (t)*.

§ *Values are analyzed by one-way ANOVA (F)*.

*The bold values mean P < 0.3 and these variables entry into multiple stepwise linear regression analysis*.

**Table 2:** Univariate analysis of clinical and psychological characteristics of body image concern in patients with acromegaly.

**Characteristics**	**Patients (*N*)**	**Total score of BICI**	**Statistical value**	***P-*** **value**
Length of hospitalization (day)^§^			2.341	**0.104**
≤ 10	27	43.48 ± 12.42		
11–14	29	48.93 ± 15		
≥15	12	53.00 ± 12.05		
Course of disease (year)^§^			1.344	**0.250**
<5	37	45.22 ± 14.90		
5–10	11	51.18 ± 13.81		
>10	20	49.65 ± 11.32		
BMI^§^			2.065	**0.135**
18.5–24.9	28	43.50 ± 16.34		
25–30	28	50.00 ± 11.12		
30–34.9	12	50.92 ± 11.52		
PRL^†^	–	–	0.223	**0.072**
ACTH^†^	–	–	0.216	**0.101**
Thyroid hormone^§^			0.366	0.695
Low	9	50.33 ± 10.45		
Normal	56	46.82 ± 16.41		
High	3	51.33 ± 2.51		
Pre-GH^†^	–	–	0.039	0.755
FBG^†^	–	–	0.123	0.320
Pos-GH^‡^			−1.24	**0.219**
Normal	31	45.23 ± 14.70		
High	37	49.38 ± 12.91		
Tumor size^†^	–	–	0.026	0.835
Recurrence^‡^			0.075	0.941
Yes	7	47.86 ± 11.09		
No	61	47.44 ± 14.16		
Comorbidity^‡^			0.673	0.503
Yes	38	48.46 ± 14.28		
No	30	46.17 ± 13.28		
Hormone Therapy after discharge^‡^			−1.098	**0.276**
Yes	47	45.00 ± 16.16		
No	21	48.84 ± 12.33		
HAMA^†^			0.548	**<0.001**
Mild anxiety	9	30.67 ± 14.81		
Moderate anxiety	15	44.67 ± 13.03		
Severe anxiety	36	50.89 ± 11.51		
Extreme anxiety	8	56.38 ± 6.35		
SSCI^†^	–	–	0.679	**<0.001**
SSCI-I	–	–	0.711	**<0.001**
SSCI-E	–	–	0.546	**<0.001**
BIPQ^†^	–	–	0.479	**<0.001**

*NFPA, nonfunctioning pituitary adenomas; BMI, body mass index; PRL, prolactin; ACTH, adrenocorticotropic hormone; FBG, fasting blood-glucose; Pre-GH, preoperative growth hormone; Pos-GH, postoperative growth hormone; BICI, Body Image Concern Inventory; HAMA, Hamilton Anxiety Scale; SSCI, Stigma scale for chronic illness; SSCI-I, internalized stigma; SSCI-E, enacted stigma; BIPQ, Brief Illness Perception Questionnaire*.

*Values are presented as number and mean ± standard deviation*.

† *Values are analyzed by Pearson correlation analysis*.

‡ *Values are analyzed by independent sample test (t)*.

§ *Values are analyzed by one-way ANOVA (F). The bold values mean P < 0.3 and these variables entry into multiple stepwise linear regression analysis*.

### Body Image Concerns in Patients With Acromegaly Compared With Patients With NFPA

**Figure 1:**
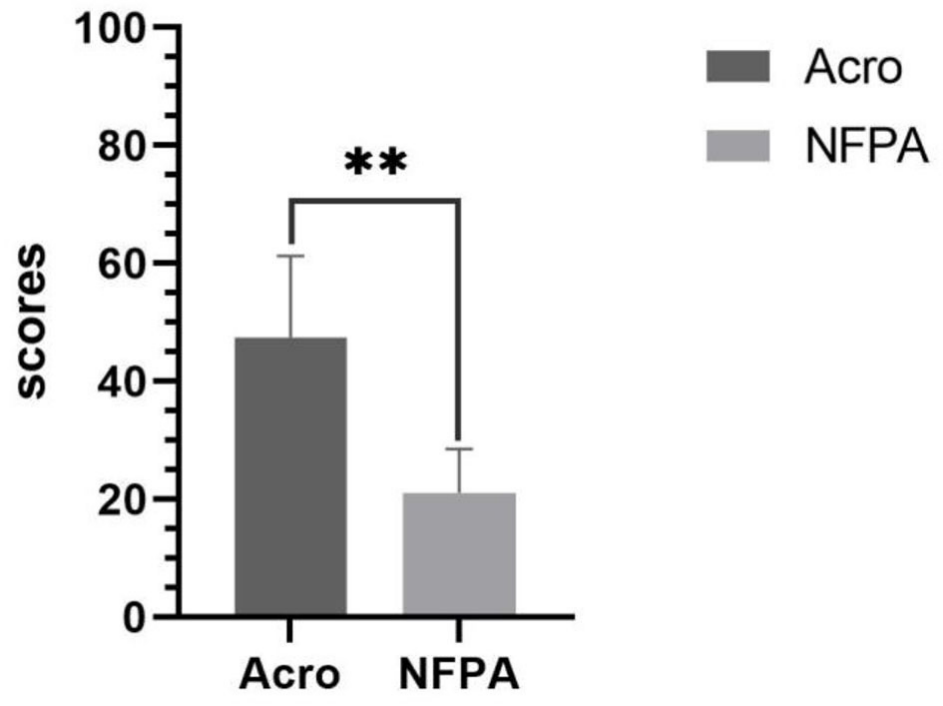
Body image concern in patients with acromegaly vs. those with NFPA. Data are presented as mean ± standard deviation. Acro, acromegaly; NFPA, nonfunctional Pituitary Adenoma. **Correlation is significant at the 0.01 level (2-tailed).

The BICI scores showed that patients with acromegaly (both men and women) reported severe body image problems compared with patients with NFPA. The mean BICI score was 47.49 ± 13.81 for patients with acromegaly and 21.10 ± 7.44 for patients with NFPA. The difference between the two groups was statistically significant (*P* < 0.001) (Figure 1).

### Correlations Between Demographic, Clinical, and Psychological Characteristics and Body Image Concerns Among Patients With Acromegaly

Spearman's rank correlations were calculated to identify the relationships among demographic, clinical, and psychological characteristics and body image concerns in patients with acromegaly. The following variables were correlated with body image concerns: age at diagnosis (*r* = −0.181, *P* = 0.139), marital status (*t* = −3.086, *P* = 0.056), profession (*F* = 1.609, *P* = 0.196), economic status (*F* = 1.729, *P* = 0.170), payment of medical expenses (*F* = 3.897, *P* = 0.025), length of hospitalization (*F* = 2.341, *P* = 0.104), course of disease (*F* = 1.344, *P* = 0.25), body mass index (*F* = 2.065, *P* = 0.135), PRL level (*r* = 0.223, *P* = 0.072), ACTH hormone level (*r* = 0.216, *P* = 101), growth hormone level after surgery (*t* = −1.24, *P* = 0.219), and hormone therapy after discharge (*t* = −1.098, *P* = 0.276).

The following correlations between psychological factors and body image were found: illness perceptions, anxiety, and stigma were closely related to body image concerns. The mean BIPQ score for patients with acromegaly was 51.56 ± 7.41 (*r* = 0.479, *P* < 0.001) and the anxiety score was 21.56 ± 6.05 (*r* = 0.548, *P* < 0.001). Patients with acromegaly generally had higher anxiety levels and negative illness perceptions, which suggests poor psychological status. The mean SSCI score was 58.78 ± 12.8 (*r* = 0.679, *P* < 0.001), the internalized stigma score was 36.57 ± 7.9 (*r* = 0.711, *P* < 0.001), and the enacted stigma score was 22.22 ± 5.55 (*r* = 0.546, *P* < 0.001), which indicated a strong sense of stigma (Tables 1, 2).

### The Results of Multiple Stepwise Linear Regression of Body Image Concerns

A multiple stepwise linear regression was performed for univariate variables with a *P*-value < 0.3 and significant variables from most previous studies (e.g., gender) were identified as the potential predictors of body image concerns in patients with acromegaly. The results showed that factors related to body image were being female (β = 0.247, *P* = 0.001), young age at diagnosis (β = −0.184, *P* = 0.01), and internalized stigma (β = 0.88, *P* < 0.001; Adj. *R*^2^ = 0.756) (Table 3).

**Table 3:** Stepwise multiple linear regression analysis per determinant for body image concern in patients with acromegaly.

	***B*** **(SE)**	**β**	***t***	**(95% CI)**	***P*** **-value**	**Model summary**
Gender	6.940 (1.947)	0.247	3.565	(3.036, 10.843)	0.001	*F* = 60.003
Age at diagnosis	−0.212 (0.079)	−0.184	−2.668	(−0.371, −0.053)	0.01	*P* < 0.001
SSCI-I	1.589 (0.120)	0.880	13.285	(1.349, 1.829)	<0.001	Adjusted *R*^2^ = 0.756

*SSCI-I, Internalized stigma*.

### Correlation Analysis Results of Body Image Concerns and Quality of Life Among Patients With Acromegaly

Table 4 presents the correlations between the BICI scores and the SF-36 scores among patients with acromegaly. The BICI score was significantly correlated with many QoL dimensions. The correlations were as follows: physical functioning (*r* = −0.201, *P* = 0.101), role-physical (*r* = −0.231, *P* = 0.058), bodily pain (*r* = −0.185, *P* = 0.131), general health (*r* = −0.265, *P* = 0.029), physical component summary (*r* = −0.214, *P* = 0.08), vitality (*r* = −0.232, *P* = 0.057), social functioning (*r* = −0.293, *P* = 0.015), role-emotional (*r* = −0.248, *P* = 0.042), mental health (*r* = −0.382, *P* = 0.001), and mental component summary (*r* = −0.346, *P* = 0.004) (Table 4).

**Table 4:** Correlation analysis of body image concern and quality of life in patients with acromegaly.

**Items**	**PCS**	**MCS**	**PF**	**RP**	**BP**	**GH**	**VT**	**SF**	**RE**	**MH**
BICI	−0.214	−0.346^**^	−0.201	−0.231	−0.185	−0.265^*^	−0.232	−0.293^*^	−0.248^*^	−0.382^**^

** *Correlation is significant at the 0.01 level (2-tailed)*.

* *Correlation is significant at the 0.05 level (2-tailed)*.

*PCS, physical component summary; MCS, mental component summary; PF, physical functioning; RP, role-physical; BP, bodily pain; GH, general health; VT, vitality; SF, social functioning; RE, role-emotional; MH, mental health*.

### Comparison of SF-36 Scores Between Patients With Acromegaly and Patients With Nonfunctional Pituitary Adenoma

**Figure 2:**
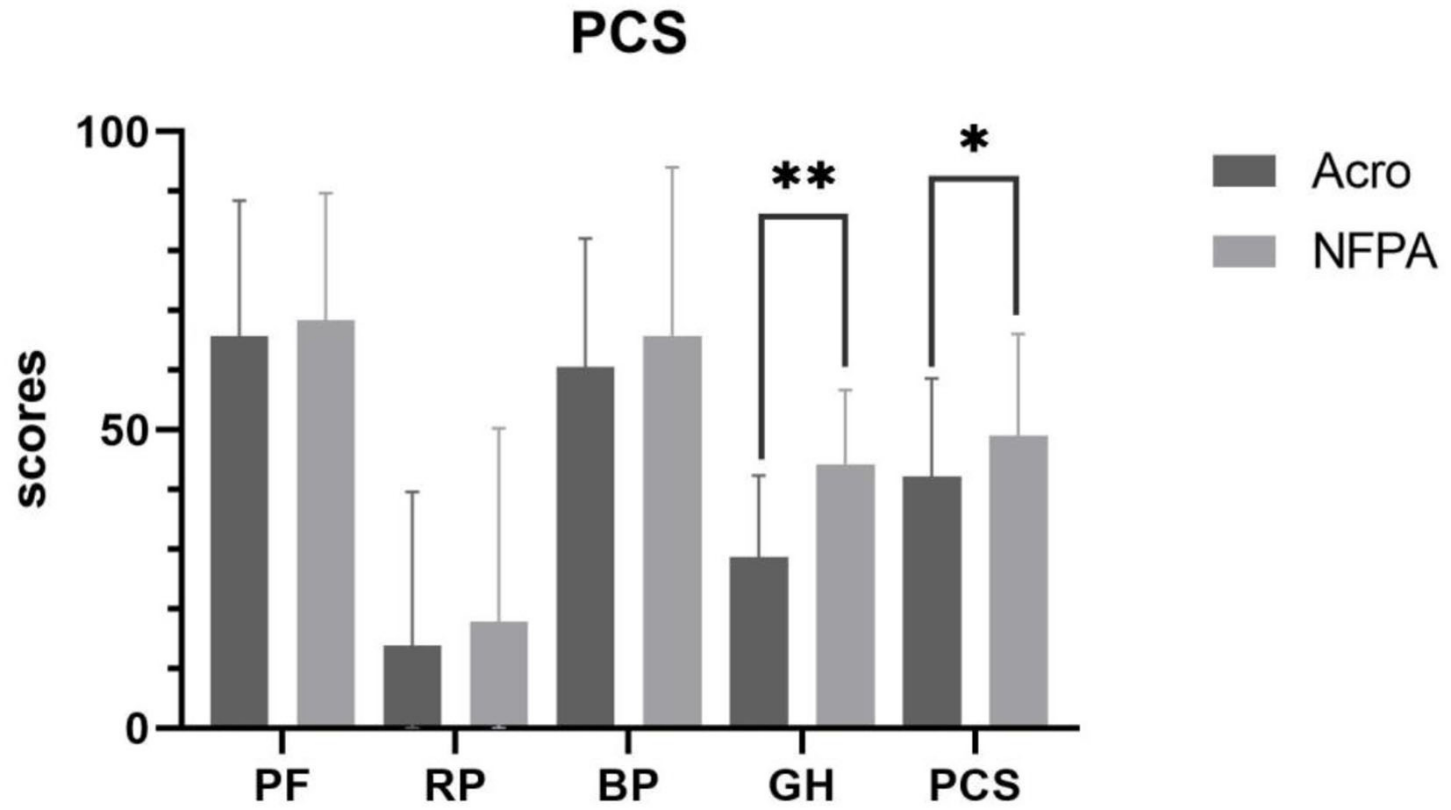
Physical component summary score in patients with acromegaly vs. those with NFPA. Data are presented as mean ± standard deviation. PF, physical functioning; RP, role-physical; BP, bodily pain; GH, general health; PCS, physical component summary. **Correlation is significant at the 0.01 level (2-tailed). *Correlation is significant at the 0.05 level (2-tailed).

**Figure 3:**
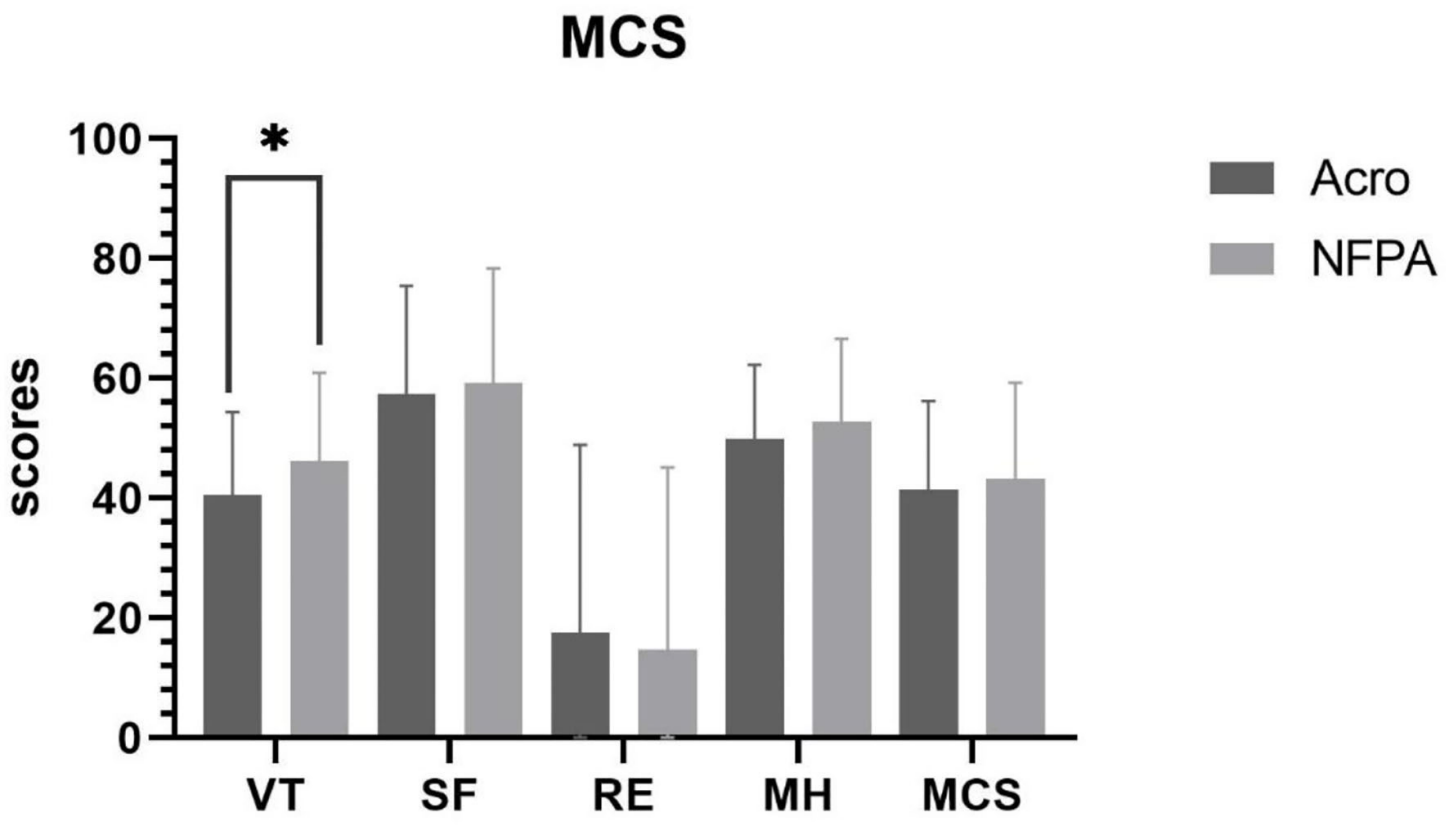
Mental component summary score in patients with acromegaly vs. those with NFPA. Data are presented as mean ± standard deviation. VT, vitality; SF, social functioning; RE, role-emotional; MH, mental health; MCS, mental component summary. *Correlation is significant at the 0.05 level (2-tailed).

The overall level of health was poorer in patients with acromegaly than in those with NFPA. The physical component summary score in patients with acromegaly was 42.27 ± 16.35, whereas it was 49.05 ± 17.05 (*P* = 0.019) in patients with NFPA. The mental component summary score was 41.36 ± 14.83 in acromegalic patients with acromegaly and 43.26 ± 16.05 in patients with NFPA. The general health scores of patients with acromegaly (28.71 ± 13.64) were lower than those of patients with NFPA (44.18 ± 12.51) (*P* < 0.001). The vitality scores of patients with acromegaly (40.51 ± 13.88) were lower than those of patients with NFPA (46.21 ± 14.71) (*P* = 0.021). However, the role-emotional scores of patients with acromegaly (17.64 ± 31.26) were higher than those of patients with NFPA (14.76 ± 30.37) (*P* = 0.583, *ns*) (Figures 2, 3).

## Discussion

In this study, we investigated the body image concerns in patients with acromegaly and patients with NFPA. The multiple stepwise linear regression results showed that factors related to body image concerns in patients with acromegaly were being female, young age at diagnosis, and internalized stigma. Additionally, we found that patients with acromegaly rated their QoL as poor compared to patients with NFPA and had substantial anxiety, negative illness perceptions, stigma, and body image concerns.

Body image is a useful psychological concept for understanding an individual's perception of their body in the context of the social environment. Acromegaly is characterized by progressive morphometric changes such as fleshy lips, acral enlargement, nose enlargement, jawbone growth, prominence of the skull and skin, and soft tissue changes, which may lead to severe disfigurement over time. The pathological mechanism that causes these body image abnormalities may be the long-term effects of GH/IGF-1 hypersecretion on multiple organs and tissues (Caron et al., 2019). Moreover, most of these changes are only partly reversible, and most patients never regain their premorbid appearance, even after the disease has been biochemically controlled. Consistent with our prediction, younger patients and female patients had more severe body image concerns. In contemporary culture, women tend to be more concerned about their body image than men (Aparicio-Martinez et al., 2019). The development of an abnormal body image caused by elevated hormone levels is difficult for patients to cope with and may result in discrimination. In the long-term care of patients with acromegaly, body image concerns could be mitigated by reducing the patient's focus on their appearance. For female patients with acromegaly, different educational guidance methods should be used that are tailored to different cultures to reduce body image concerns and feelings of inferiority. This is a long-term process that requires the guidance of a professional psychologist and could be provided as part of the patient's continuing care. It may also be feasible to implement such guidance in community care.

Our findings are generally consistent with previous research on body image concerns in patients with acromegaly (Conaglen et al., 2015). However, our findings differ from those of previous studies regarding the association between anxiety, depression, and body image concerns (Dimopoulou et al., 2017). Previous studies suggest that body image perception in acromegaly correlates with depressive symptoms. Although we found a close correlation between anxiety and body image concerns, anxiety did not appear to be a predictor of body image concerns in the stepwise multiple regression analysis. However, patients' anxiety cannot be ignored. We need to pay close attention to anxiety and body image issues, as increasing numbers of people experience anxiety about abnormal body image (Bekhuis et al., 2016). Interventions to address patients' body image concerns may help to relieve anxiety about body image.

Acromegaly is an endocrine disorder characterized by different body impairments and deformities. Patients are susceptible to stigma because of the disease's gradual onset and chronic nature, as well as its permanent effects and complications. It is noteworthy that patients with acromegaly experience moderate stigma and that internalized stigma is associated with body image concerns. The internalized cognitive, behavioral, and emotional effects of others' unfavorable attitudes toward a person with devalued characteristics are known as self-stigma (Rusch et al., 2005). According to Corrigan's self-stigma theoretical model, self-stigma is a process in which community perception leads to personal reactions and, eventually, self-stigma (Steward et al., 2008). Research on disease stigma has increased in recent years, but there are few studies on patients with acromegaly or those with PA. Previous studies have focused on patients with body image changes similar to those with acromegaly, such as colostomy, obesity, and cancer. Acromegaly belongs to two parts of the three major categories of stigma research: diseases with altered appearance and chronic diseases. Therefore, its stigma deserves more attention. Additionally, the internalized stigma of patients with acromegaly is serious, suggesting that patients' stigma stems from self-perception, and patients may have inferior personality and psychological distress, which are also closely related to patients' body image concerns (Hatzenbuehler, 2016). These patients also experience severe stigma, accompanied by poor QoL (Jin et al., 2020; Huang et al., 2021). Therefore, the effectiveness of reducing patients' body image concerns by reducing stigma needs to be further explored. Exercise may have a positive effect on patients with acromegaly' QoL and body-self-image. Research shows that after participating in a fitness routine, the self-assessment of body image substantially improved. Therefore, exercise may be an additional approach that could reduce body image concerns and boost confidence in patients with acromegaly. Such methods should be actively implemented (or improved) for patients with severe internal stigma to try to change their cognition, to reduce stigma, and to ultimately alleviate their body image concerns.

It is worth noting that, combining the findings of the literature and clinical experience (Tiemensma et al., 2010; Zimmermann et al., 2018; Wolters et al., 2020; Guo et al., 2021), we also hypothesized at the beginning of the study that there was a correlation patients ' between hormone levels and psychology. However, only PRL, ACTH, and postoperative growth hormone levels were statistically significant in the univariate analysis, and the results of multiple linear regression showed no association between hormone levels and psychology. Consistent with the previous studies (Imran et al., 2016; Zimmermann et al., 2018), we cannot determine the exact relationship between hormone levels and psychology in patients. The findings of previous scholars are inconsistent, for instance, Guo's research showed that hormone levels were not associated with patients' psychology (Guo et al., 2021). However, Sievers's research had shown that psychiatric symptoms overlap with periods of GH overproduction (Sievers et al., 2009a). Possible explanations for these findings may be related to alterations in the macroscopic brain architecture with increased global, left, and right hippocampal gray matter and white matter volumes at the expense of cerebral spinal fluid (Sievers et al., 2009b, 2012), as demonstrated in cerebral MRI. In addition, while hormone levels returning to normal would not make the patient's facial features completely return to their pre-acromegalic state, there would be an improvement in facial features, hand and foot size, and other metabolic effects, and that would make the patient feel more comfortable in society. However, some studies have shown that the psychological changes in patients with acromegaly are not limited to the time of hormonal abnormalities but still persist in cured or controlled patients (Zimmermann et al., 2018). Therefore, we will continue to follow up patients, expand the sample size, and measure hormone levels at more time points in the next stage of the study to analyze the influence of hormone levels on patients' body image concerns and other mental health factors.

Finally, the present findings demonstrate that patients with acromegaly have body image concerns, and these concerns may explain why such patients have poor QoL. Body image concerns in patients with acromegaly were negatively correlated with the following aspects of QoL: social functioning, general health, emotional roles, and mental health. Significant differences were found between patients with acromegaly and patients with NFPA on the QoL aspects of general health, vitality, and physical component summary. This finding may indicate that patients with NFPA have poor QoL (Butterbrod et al., 2019; Pertichetti et al., 2020); poor mental health is a common problem in such patients. Patients with acromegaly had low physical component summary scores.

This is consistent with the musculoskeletal discomfort, acral overgrowth, arthritis (Miller et al., 2008), soft tissue swelling, and fatigue (Rowles et al., 2005) reported by patients with acromegaly in this and other studies. Patients with acromegaly are more likely to experience many complications, and so have a poorer physical function than patients with NFPA (Colao et al., 2004). In recent years, the importance of psychosocial functioning in patient-centered healthcare has been growing. QoL, body image, self-esteem, and other psychological constructs are increasingly investigated as criteria to evaluate the benefits of drug therapy (Shpigelman and HaGani, 2019). Therefore, we should pay special attention to general health in patients with acromegaly and physical function problems. We may need to improve patients' overall health to enhance their QoL. Previous studies have shown that appropriate postoperative cognitive–behavioral treatment and sustained drug therapy are helpful in improving health (Wild et al., 2020). A combination of these two treatments may be worth considering in the rehabilitation of patients. From the perspective of nursing, patients need to receive appropriate psychological nursing or psychological guidance, and medication and treatment compliance in patients must be improved. This may help to reduce patients' body image concerns and improve their QoL.

This study had both strengths and limitations. The study is somewhat innovative because previous studies exploring factors related to body image issues in patients with acromegaly are relatively scarce, compared with studies of other medical conditions that involve body disfiguration (e.g., cleft lip patients, breast cancer survivors, anorexia nervosa patients). A total of 68 individuals with acromegaly were enrolled in our study. A total of 70 persons with NFPA were randomly recruited as a healthy control group. Using structured questionnaires, such as BICI, we explored perceived body image. We also used HAMA, SSCI, BIPQ, and SF-36 to evaluate the health-associated conditions and analyze the related factors that influence the body image concerns in patients with acromegaly. People with acromegaly have serious body image concerns. The stigma around the disease also makes sufferers more concerned about their image. Such worries lead to poor QoL. Doctors may need to find better ways to control their patients' hormone levels, and the nurses should provide more information on how to improve the patient's body image or find ways to reduce the patient's concerns with body image. The study limitations are as follows: first, the cross-sectional design restricts our ability to make causal inferences about acromegaly-related abnormalities and the development of body image concerns. In future studies, we will further analyze the causal relationship between psychological factors and clinical indicators of acromegaly through RNA sequencing and Mendelian randomization analysis (Hou et al., 2020; Wang et al., 2021; Zhang et al., 2021). Second, our results do not yet determine the effect of hormone levels on patients' psychology. We will continue to follow up with patients, measuring hormone levels at more time points in the next stage of the study to analyze the relationship between patients' hormone levels and psychology. Third, we only described and briefly compared the body image and QoL of patients with acromegaly and control patients, which makes it difficult to analyze the causal relationships between the variables in any detail. Fourth, this study was not a single-center study, and the number of cases was limited, so the results may not be a representative of all patients with acromegaly. A multi-center study should be carried out in the later period to obtain a sufficient sample size with multiple characteristics. These shortcomings should be improved in patient follow-up and future studies.

## Conclusions

To summarize, patients with acromegaly have a poor body image that is not related to anxiety but is closely associated with stigma. The stigma surrounding this disease also increases patients' concerns about their body image. Such concerns lead to poor QoL. Physicians need to find better ways to control patients' hormone levels, and nurses should provide more information on how to improve patients' body image or find ways to reduce patients' concerns about body image. More importantly, nurses need to pay more attention to patients' psychological distress and invite professional psychologists to evaluate and diagnose patients' mental status. Psychosocial care should be administered under the guidance of a professional psychologist.

## Data Availability Statement

The original contributions presented in the study are included in the article/supplementary material, further inquiries can be directed to the corresponding author/s.

## Ethics Statement

The experimental protocol was established, according to the ethical guidelines of the Helsinki Declaration. Ethical permissions were granted by the Human Ethics Committee at Affiliated Hospital of Nantong University (No. 2018-K020). Written informed consent was obtained from individual or guardian participants. Written informed consent for participation was not required for this study in accordance with the national legislation and the institutional requirements.

## Author Contributions

XZ participated in data visualization, conceptualization, and writing—original draft and formal analysis. YL participated in formal analysis and data curation. YZ participated in conceptualization, gathering resources, review and editing, supervision, project administration, and funding acquisition. ZW participated in conceptualization, investigation, writing—review and editing. All authors contributed to the article and approved the submitted version.

## Funding

The research was supported by the Jiangsu Modern Hospital Management Research Center (JSY-3-2019-067) and the Science Foundation of Nantong City Grant (Grant Nos. MS12020011 and MSZ18100).

## Conflict of Interest

The authors declare that the research was conducted in the absence of any commercial or financial relationships that could be construed as a potential conflict of interest.

## Publisher's Note

All claims expressed in this article are solely those of the authors and do not necessarily represent those of their affiliated organizations, or those of the publisher, the editors and the reviewers. Any product that may be evaluated in this article, or claim that may be made by its manufacturer, is not guaranteed or endorsed by the publisher.
